# Vitamin D and adolescent idiopathic scoliosis, should we stop the hype? A cross-sectional observational prospective study based on a geometric morphometrics approach

**DOI:** 10.1007/s00586-023-07566-y

**Published:** 2023-02-11

**Authors:** José María González-Ruiz, Markus Bastir, Javier Pizones, Carlos A. Palancar, Viviana Toro-Ibacache, María Dolores García Alfaro, Lucía Moreno Manzanaro, José Miguel Sánchez Márquez, María Isabel Pérez Núñez

**Affiliations:** 1 Paleoanthropology Group, Museo Nacional de Ciencias Naturales, CSIC, J.G. Abascal 2, 28006 Madrid, Spain; 2 Spine Unit, Orthopaedic Surgery Department, Hospital Universitario La Paz, Madrid, Spain; 3 Institute for Research in Dental Sciences, Faculty of Dentistry, Universidad de Chile, Santiago, Chile; 4 Department of Orthopedic Surgery and Traumatology, University Hospital of Valdecilla, ESQUEMA Group IDIVAL, University of Cantabria, Santander, Spain

**Keywords:** Adolescent idiopathic scoliosis, Fluctuating asymmetry, Vitamin D, Cobb angle, Torso

## Abstract

**Purpose:**

There is strong evidence supporting the presence of fluctuating asymmetry (FA) in Adolescents with Idiopathic Scoliosis (AIS). Additionally, recent research investigating the relationship between vitamin D and AIS found a relation between them. We hypothesize a negative correlation between FA and vitamin D.

**Methods:**

We performed a surface scan of the torso of 53 AIS patients, a blood test to measure vitamin D and the radiographic Cobb angle. A correlation analysis between vitamin D and FA was carried out to test our hypothesis, and a regression of vitamin D on 3D shape was performed to observe shape differences between the vitamin D deficiency and insufficiency groups.

**Results:**

There was no correlation between vitamin D and FA. We found a strong negative correlation between vitamin D and the Cobb angle only in the premenarche group (*n* = 7; *r* = − 0.92). Differences in shape were observed between the deficiency and insufficiency groups, and that differences were related to the width of the torso, but not the rotation or lateral flexion.

**Conclusions:**

Our results do not support the massive screening of vitamin D in AIS. Shape analysis revealed differences between the shape of the deficiency and insufficiency groups related to robustness. However, this finding had no relation with the scoliosis characteristics, it just reflected different body composition, and its importance should be explored in future.

## Introduction

Fluctuating asymmetry (FA) is a biological phenomenon that comprises all random and small magnitude asymmetries in a hypothetically symmetrical shape during growth [1, 2]. In biological research, FA has been considered a powerful biomarker of developmental instability [3, 4]. In this manner, individuals with higher values of fluctuating asymmetry are likely to have undergone greater instability during development due to environmental factors. In the case of idiopathic scoliosis, these environmental factors could be related to epigenetic mechanisms such as nutrition, life style, vertebral growth plates asymmetries, quality of vertebral bone or sagittal factors among others [5], and the resulting phenotypic asymmetries will be present in patients during their adult life [6]. Thus, the quantification of the individual fluctuating asymmetry levels in patients with adolescent idiopathic scoliosis [AIS] is a promising tool to assess the magnitude of developmental instabilities during growth [7]. In this developmental period, the AIS condition is in its higher risk of progression, reducing the individual´s growth quality [8, 9]. Once the presence of FA has been evidenced in patients with AIS [7, 10–12], we need to further investigate its role in the development of the scoliotic phenotype during adolescence.

A recent study has suggested a link between vitamin D and AIS due to the key role played by the former in bone metabolism during skeletal development [13]. In a systematic review, although with low level of evidence [14], the authors found that patients with AIS had lower seric levels of vitamin D than controls, independent of sex or menarche presence. As a conclusion, they recommended to screen vitamin D levels in AIS patients, since it may be involved in the pathogenesis of the condition by affecting the calcium-phosphorus bone metabolism, although the calcium levels were normal in their sample. Similar conclusions were presented by Balioglu et al. [18], where a statistically significant negative correlation between vitamin D status and Cobb angle was found. However, this correlation between Cobb angle, the main radiological variable of scoliosis severity, and vitamin D levels was not found in a recent research by Alsiddiky et al. [15], despite the high prevalence of vitamin D insufficiency observed among the AIS patients included in the study (92%). According to different experts, less than 30 ng/mL of vitamin D corresponds to insufficiency, and less than 20 ng/mL is considered deficiency [13, 16, 17] (US Endocrine Society, https://www.endocrine.org/).

Additionally, although the relationship between low level of vitamin D and AIS has been recently evidenced, the actual role of vitamin D in AIS etiophatogenesis remains unclear. New theories that link insufficiency and deficiency of vitamin D with bone metabolism, which has been found in patients with AIS, are attracting attention of scoliosis researchers [18]. Even its role in the muscle physiology must be considered, due to its participation at skeletal support and motor development in these patients [18].

With all this background we aimed to test the hypothesis of association between lower levels of vitamin D serum level and higher individual FA score. Simultaneously, it is expected to found a relationship between the worst cases of AIS, measured in terms of Cobb angle and tridimensional shape of the torso, with higher FA scores and lower vitamin D serum values.

## Methodology

### Sample description and ethical statement

This is a cross-sectional observational prospective study involving two hospitals. 53 AIS patients (16 from one center, and 37 from the other) matched the inclusion criteria, which were the following: both genders, age between 10 and 16 (both included), Cobb angle greater than 20° and Tanner scale of 4 or less. Exclusion criteria were previous vitamin supplementation, chronic disease (particularly celiac disease, liver failure, diabetes or endocrine disorders of calcium-phosphorous metabolism), fractures during the two months previous to the beginning of the study, or treatment based on antiepileptic drugs and steroids.

The study protocol was approved by the Ethics Committee of Medical Investigation of both hospitals with code 2021.183 (28th of May, 2021). We have conducted all the study following the protocols described in the Helsinki Declaration for human-based research.

### Surface data acquisition

We used an Artec™ MHT 3D scanner to surface scan the torso of the 53 patients following our previous protocol described in [19]. All PLY files were digitized in http://www.dhal.com/viewbox.htm following the template previously described [19] and the shape coordinates from the digitization process were used in further analysis.

### Blood analysis and medical variables acquisition

The radiographic variables examined were the Risser stage and the Cobb angle of the main and secondary curves. Blood analyses included the following variables: calcium, phosphate, magnesium, albumin, vitamin D and PTH. Normal values expected are the following: calcium (9.1–10.2 mg/dL), phosphate (2.5–4.5 mg/dL), magnesium (1.6–2.6 mg/dL), albumin (3.8–5.4 g/dL), vitamin D (30–75 ng/mL) and PTH (18.5–88 pg/mL). Vitamin D variable was measured as 25-hydroxyvitamin D (25(OH)D). All these measurements together with the scanning of the torso were obtained in the same visit to minimize confounders. Visits were performed between October 2021 and February 2022 to obtain accurate measurements of Vitamin D levels not influenced by sun exposure.

### Statistical analysis of shape and statistics

We used the reference analytical values to screen for abnormal cases, which were found in vitamin D (below 30 ng/mL), calcium (above 10.2 mg/dL) and phosphate (above 4.5 mg/dL). Vitamin D, calcium and phosphate were correlated between each other and with the main variables of the study: main Cobb angle and FA score.

Then, we performed a Procrustes ANOVA analysis in MorphoJ [20], which is a two-way ANOVA analysis of the Procrustes shape coordinates of the 53 patients, to test for the presence of FA in the sample [21], which needs to be compared against the measurement error introduced by landmarking digitization. To quantify measurement error, sample data were digitized twice following the recommendations from Fruciano [22]. The individual FA scores, which are the individual Procrustes distance to the mean asymmetry of the sample [7] were exported to test the main hypothesis.

Posterior analyses were conducted with the whole sample using Past 4.03 (https://palaeo-electronica.org/2001_1/past/issue1_01.htm) and, then separating the sample into groups taking into consideration the following factors related to vitamin D absorption: pre-menarche and post-menarche groups (males excluded), latitude above or below 42° north [23] and sex. A *t*-test or a Mann–Whitney test was used to test mean or median differences depending on the data distribution. A *p* value < 0.05 was considered statistically significant to test differences between groups [pre- and post-menarche, latitude above or below 42° north and sex] for the variables Vitamin D, FA score, main and secondary Cobb angle.

The next step was the correlation analyses, that were carried out between the 3 reference variables (Cobb angle, vitamin D and individual FA score) with no assumptions of dependence. A Pearson correlation was used when the 2 variables followed the normal distribution, and a Spearman´s D was used in the case that both, or at least one of the variables, did not follow the normal distribution. Additionally, some of the correlations carried out by groups (latitude, menarche and sex) needed a Spearman´s rs correlation due to small sample sizes equal or less than 9.

To analyze if the asymmetric component of the shape of the torso was related with vitamin D, we did a multivariate multiple regression analysis between 3D shape and vitamin D. We explored this analysis using latitude group (below and above 42°), menarche presence and sex.

Finally, we carried out a principal component analysis (PCA) of the shape coordinates in MorphoJ [20] to represent in orthogonal axes the shape variability of the sample. Then, the PC scores of the PCA that accumulate up to the 75% of the variability, were used as shape variables to test differences between groups (deficiency, insufficiency and sufficiency of vitamin D) using a one-way PERMANOVA test with 9999 permutations. Post hoc analyses were conducted to screen for significant differences between groups.

## Results

### Blood test analyses and medical variables

Mean age of the sample was 14.18 (± 1.3) with a sex distribution of 15.1% males (*n* = 8) and 84.9% females (*n* = 45).

Table 1 shows specific variables that characterized the sample, expressed as mean and SD for quantitative variables and median and interquartile range for qualitative variables.

**Table 1:** Specific variables expressed as mean and standard deviation (SD) for Cobb angles and median and interquartile range-IQR-(25–75) for Risser

Risser Median (IQR)	Cobb angle of main curve Mean (± SD)	Cobb angle of secondary curve Mean (± SD)
3 (1–4)	35.13 (± 11.49)	30.67 (± 11.44)

Mean values and SD of blood analysis variables were the following: calcium 10.14 (0.36), phosphate 4.35 (0.53), magnesium 1.97 (0.18), albumin 4.77 (0.24), vitamin D 23.17 (6.8) and PTH 46.97 (18.98). 18 patients (33.96%) had hypercalcemia and 7 (13.2%) presented hyperphosphatemia. In terms of sample distribution, 16 patients (30.19%) had deficiency (below 20 ng/mL), 30 patients (56.6%) had insufficiency (between 20 and 30 ng/mL) and only 7 patients (13.21%) had sufficiency (above 30 ng/mL).

We have found a mean Vitamin D value of 23.17 (6.8), corresponding to insufficiency levels. The rest of analytical variables were normal in their mean values, but calcium and phosphate had a subgroup of patients with higher levels than normally expected.

In the group of patients with normal calcium level (9.1–10.2 mg/dL) a significant negative correlation was found between calcium and vitamin D (*p* value = 0.04), and calcium and FA score (*p* value = 0.02). No correlation was found between calcium and Cobb angle or between vitamin D, Cobb angle and FA score in these patients. In the group of patients with hypercalcemia, we did not find correlation between calcium levels and vitamin D or FA score. However, a significant positive correlation was found between calcium and Cobb angle (*p* value = 0.03), and between Cobb angle and FA score (*p* value = 0.03). No correlation was found between vitamin D and Cobb angle or vitamin D and FA score in the group of patients with hypercalcemia. The mean phosphate value was 4.77 (0.39) in the premenarche group and 4.19 (0.39) in the post-menarche group. There was no correlation between calcium and phosphate in pre-menarche and post-menarche groups, nor between phosphate and vitamin D, Cobb angle and FA score.

### Preliminary analyses

We have observed a statistically significant presence of FA in the sample (*p* value < 0.001). Also, measurement error magnitude (mean square = 1.4668 × 10^–6^) was lower than FA effect (mean square = 3.0669 × 10^–6^).

Table 2 shows mean and median differences for the variables vitamin D serum level, FA score, main Cobb angle and secondary Cobb angle (in patients with double curve). Results are shown by group classifiers as follows: latitude below or above 42°, pre- or post-menarche status and sex.

**Table 2:** Mean; 95% confidence interval [CI(95%)] for normal distributed samples, median; interquartile range(IQR:25–75) for nonparametric distributed samples

Classifiers	Latitude		Menarche		Sex	
Variables	38°–42°	42°–44°	No	Yes	Male	Female
Vitamin D	22.71; CI(95%) = 20.18–25.25	24.06; CI(95%) = 21.27–26.85	25.57; CI(95%) = 19.26–31.89	22.71; CI(95%) = 20.53–24.9	20.5; IQR(18–30.25)	23; IQR (18–28)
FA score	0.027; IQR (0.022–0.036)	0.026; IQR (0.022–0.032)	0.029; IQR (0.023–0.046)	0.027; IQR (0.023–0.033)	0.024; IQR (0.019–0.028)	0.027; IQR (0.023–0.035)
Main Cobb	36; IQR (28–43)	30; IQR (27–34.5)	24; IQR (20–41)	33; IQR (28–41.25)	35.5; IQR (27.75–45)	32; IQR (27.5–41)
Secondary Cobb	34; IQR (28.5–39.5)	22; IQR (17.75–27.75)	**20; IQR (17–20)**	**31; IQR (23–35.5)**	34; IQR (24.5–43.5)	29.5; IQR (22–34.75)

Bold cells show significant differences between groups (*p* value < 0.05)

### Hypothesis test

The results of the correlations between vitamin D, main and secondary Cobb angles and FA scores are shown in Table 3. All correlations were carried out on the whole sample and by latitude, menarche and sex classifier. A negative significant correlation was found between vitamin D and main Cobb angle in the premenarche state and between vitamin D and secondary Cobb angle in latitude below 42°. A positive significant correlation was found between FA score and main Cobb angle in the whole sample, in latitude below 42°, in post-menarche and in the female group.

**Table 3:** Spearman correlations were represented by Statistic (Stat.) and *p* value

Classifiers	Whole sample	Latitude		Menarche		Sex	
		38°–42°	42°–44°	No	Yes	Male	Female
*N*	53	35 and 21 (Cobb2)	18 and 12 (Cobb2)	7 and 3 (Cobb2)	38 and 25 (Cobb2)	8 and 5 (Cobb2)	45 and 28 (Cobb2)
Vitamin D—FA score	Stat. = 25,274 *p* value = 0.88	Stat. = 8319.5 *p* value = 0.33	*r* = 0.33 *p* value = 0.18	*r* = − 0.73 *p* value = 0.06	Stat. = 9230 *p* value = 0.94	Stat. = 0.66 *p* value = 0.08	Stat. = 17,049 *p* value = 0.41
Vitamin D—main Cobb	Stat. = 26,527 *p* value = 0.6	*r* = − 0.08 *p* value = 0.65	Stat. = 932.5 *p* value = 0.9	***r* = − 0.92** ***p* value = 0.01**	Stat. = 8831.5 *p* value = 0.85	Stat. = 0.49 *p* value = 0.22	Stat. = 18,018 *p* value = 0.21
Vitamin D—secondary Cobb	Stat. = 7640 *p* value = 0.11	***r* = − 0.45** ***p* value = 0.04**	Stat. = 284 *p* value = 0.99	Stat. = 0 *p* value = 1	Stat. = 3239.5 *p* value = 0.22	Stat. = − 0.15 *p* value = 0.83	Stat. = 4699.5 *p* value = 0.13
FA score—main Cobb	**Stat. = 16,159** ***p* value = 0.01**	**Stat. = 3685.5** ***p* value = 0.01**	Stat. = 836.5 *p* value = 0.58	*r* = 0.62 *p* value = 0.14	**Stat. = 5930** ***p* value = 0.03**	*r* = 0.34 *p* value = 0.41	**Stat. = 9573.5** ***p* value = 0.01**
FA score—secondary Cobb	Stat. = 4843 *p* value = 0.28	Stat. = 1194.5 *p* value = 0.32	Stat. = 194.5 *p* value = 0.29	Stat. = − 0.87 *p* value = 0.67	Stat. = 1770 *p* value = 0.12	*r* = 0.23 *p* value = 0.7	Stat. = 2700 *p* value = 0.18

Pearson correlations were represented by correlation coefficient (*r*) and *p* value. Sample size for secondary Cobb (Cobb2) is lower because not all patients have double curve. Bold cells show significant correlations

**Fig. 1:**
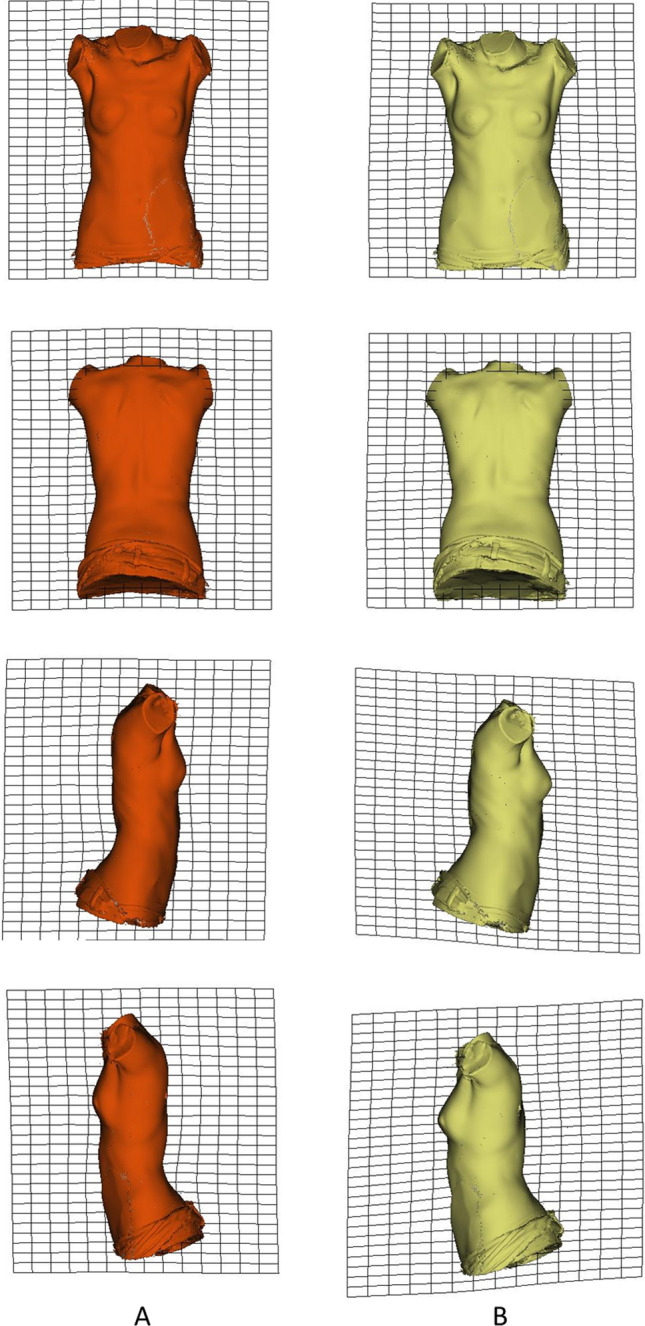
**a** From top to bottom: anterior, posterior, right, and left views of the mean shape of the deficiency group (red).** b** From top to bottom: anterior, posterior, right, and left views of the mean shape of the insufficiency group (yellow). Thin-plate spin transformation grids are shown in** a** and** b** for better visualization

None of the regression analyses of the asymmetry of shape on vitamin D were significant. Finally, PC 1 to PC 6 from PCA showed a cumulative 75.25% of variability in the sample. They were used as shape variables on the one-way PERMANOVA test with a result of *F* = 2.08 (*p* value = 0.04). Post hoc analysis showed significant differences between insufficiency and deficiency groups (*p* value = 0.04). Then, the mean shape coordinates of both groups were interpolated in EVAN Toolbox 1.71 for descriptive comparison. Figure 1 shows comparison between groups using deformation grids. No differences were observed regarding inclination or rotation deformities, but a narrower torso in the frontal and sagittal plane was observed in the deficiency group. A thoracic hypokyphosis has been evidenced in the deficiency group.

## Discussion

### Blood test analysis and medical variables

Goździalska et al. [13] (15 and 18.04 ng/mL in pre- and post-menarche), Balioglu et al. [18] (17.13 ng/mL) and Alsiddiky et al. [15] (10.92 ng/mL) had found vitamin D levels that are expressions of deficiency in patients with AIS. Comparing our mean vitamin D values with the ones reported in the literature, we have found slightly higher mean levels, although they still fall into the insufficiency group (23.17 ng/mL). The prevalence of vitamin D deficiency in our sample was 30.19%, a 56.6% of the sample had vitamin D insufficiency and only the 13.21% of AIS patients had a normal level of vitamin D (> 30 ng/mL). There are two reasons that could explain this difference. First of all, it could be related to latitude of the sample residence, because it is well known that the higher the latitude, the lower the synthesis of vitamin D due to the reduction of solar radiation, especially during winter. Our study has been performed during the winter season to exclude the effect of sun exposure. Secondly, which has not been addressed in any of the studies (ours included), is the time expended by children and adolescents outdoors [24]. This is a difficult variable to assess, but relevant enough to be addressed in future studies.

All the previous studies have found a mean vitamin D value below the reference for normality (30 ng/mL), being the level of vitamin D specially lower in premenarcheal girls [13, 25]. In other study run on surgical AIS patients, only a 7.5% of the sample showed a vitamin D level above the reference value [15]. However, the prevalence of hypovitaminosis among non-scoliotic children and adolescents is also high [26] and previous studies about the relation of vitamin D and AIS based their conclusions of significant differences between AIS and control groups [13, 18]. It is well known the role of vitamin D in bone metabolism, but it is not clear yet the magnitude of the deficiency or insufficiency needed to cause clinical manifestations. Some authors have not found a relationship between vitamin D and fractures in children and adolescents [27], although in a study of 50 children who did not ingest milk (a traditional source of calcium and vitamin D) they have shown an 87% increase in fractures before the age of 7 [28]. We still do not know if vitamin D deficiency or insufficiency are enough to cause idiopathic scoliosis. In fact, if we could expect a strong role of hypovitaminosis D on bone metabolism, it should be found among patients with deficiency, that in our sample was only 30.19% of the cases. A higher prevalence of deficiency has been found in other studies [13, 17] that could justify the interpretations of the authors about the relation between vitamin D and AIS.

Our blood analysis has shown abnormal values in calcium and phosphate serum levels. Specifically, the group of patients with hypercalcemia, has shown a positive correlation with the Cobb angle. The presence of elevated values of serum calcium could be a sign of a reduction of bone calcium depot [29] that may need to be screened in patients with AIS. Nevertheless, we have only found correlation between calcium and vitamin D levels in patients with normal calcium levels, as Balioglu et al. had reported [18]. This negative correlation explains that the higher the presence of serum vitamin D, the higher the increment of bone calcium depot. In the same line of Goździalska et al. [13], we found higher phosphate levels among the premenarche group, but without correlation with vitamin D, FA score or Cobb angle. We have also found a mean PTH of 47.64 pg/mL in the post-menarche group and 41.84 pg/mL in the premenarche group, contrary to Goździalska et al. [13] but no correlation was found between PTH and vitamin D in our sample.

### Preliminary analyses

Regarding the Procrustes ANOVA analysis, we have evidenced the presence of FA and this finding is in the same line as the one reported in previous studies [7, 10–12]. Thus, AIS presents an asymmetric shape of the torso and is the result of developmental instability during growth.

### Hypothesis test

We reject our hypothesis because no correlation was observed between Vitamin D and FA. FA, as an expression of developmental instability, is the result of multiple events in AIS patients, being the multifactorial approach the accepted ethiopathogenical model [5].

However, we have found a significant correlation between FA scores and main Cobb angle, as in González-Ruiz et al. [12]. This correlation was also present in females and not in males, and in post-menarche group and not in premenarche. Considering the Cobb angle as the 2D phenotypic result of FA processes, probably in the premenarche group, where the puberty growth spurt has not yet occurred, FA has not been decisive enough to show correlation with Cobb angle.

A strong negative correlation (*r* = − 0.92) has been observed between vitamin D and the main Cobb angle in the premenarche group (*n* = 7), clearly stronger than in Balioglu et al. (*r* = − 0.15) in a higher sample that includes both sexes and menarche status (*n* = 229). It is accepted that a delay in menarche is a risk factor of AIS [30] but in our sample, vitamin D level was slightly higher in the premenarche group than in post-menarche (25.57 ng/mL and 22.71 ng/mL respectively), oppositely to the previous studies where the lowest vitamin D level has been found among premenarche girls [13, 25].

Considering that the premenarche group showed this correlation, and that they have not evidenced yet a correlation between FA and Cobb angle at this stage of maturity, we may underline the importance of screening vitamin D in this period rather than in post-menarche, where hypothetically, vitamin D deficiency could have caused an increment of FA and Cobb angle. Nevertheless, the limited sample size of premenarche patients suggest considering this result with caution.

Besides, there was no correlation between the asymmetry component of the shape of the torso and vitamin D. Even though vitamin D could hypothetically affect spine shape, the expression of this spine deformity on the 3D shape of the torso would be reduced due to soft tissues morphology (muscles, trunk fat, breast in females,…). It is well known that 3D morphology of the torso does not reflect exactly the severity of the spine curvature [12, 31, 32].

Nevertheless, qualitative shape differences were still observed between deficiency and insufficiency groups. Those differences were present in the width of the torso both in the frontal and sagittal planes, being the mean shape of deficiency group narrower than insufficiency group. According to this result we cannot support that vitamin D plays a direct role in scoliosis physiology (inclination, rotation), but it may be probably involved in the torso robustness. However, a thoracic hypokyphosis has been observed in deficiency group, which is a clinical sign of AIS. Vitamin D plays multiple functions, and in the musculoskeletal system, it affects not only bone metabolism, but skeletal muscle structure too; this effect could be a factor underlying variations in torso robustness. A reduction in muscle mass had been evidenced in subjects with low level of vitamin D [18, 33], and this could explain our finding. Additionally, there are authors who indicated that vitamin D is associated with an increase in muscle strength, which was improved through 1,25 (OH)2D (the active metabolite of vitamin D), which binds to a specific receptor of vitamin D in muscle and leads to protein synthesis and muscle cell growth [34]. We suggest exploring further the effects that hypovitaminosis D could have in skeletal muscle development, which is one of the theories included in the epigenetic-exposome theory defended by Burwell et al. [5].

Regarding the limitations of our study, although many variables have been controlled, others stayed out of our control (i.e., genetic susceptibility, hormones, exercise and nutrition,…) [35–37]. Secondly, although vitamin D could be accepted as an ethiopathogenical factor in AIS by increasing the FA score, the serum vitamin D level (probably severe deficiency) and the duration of the hypovitaminosis status (probably years, especially during growth spurts as puberty) needed to be addressed in future. Additionally, this prospective study was carried out during the Covid-19 pandemic time, where the exposition to sun radiation was significantly reduced in the population. It was also a limitation the absence of reference values of vitamin D among healthy adolescents to made possible the comparison with our observations in AIS patients.

Finally, our study has two strengths. This is the first work that investigates prospectively the correlation between vitamin D and FA, both factors previously evidenced in other studies performed on AIS. The second strength is that, to our knowledge, this is the first study that investigates the relation between vitamin D and the 3D phenotype of the torso in patients with AIS. So far, researchers had put the focus, mainly, on the relation between vitamin D and Cobb angle. Considering that AIS is a tridimensional condition we believe that more studies on the 3D aspects of shape in these patients are needed to enhance the comprehension of the condition and its etiology.

## Abbreviations

FA: Fluctuating asymmetry
AIS: Adolescent idiopathic scoliosis
PTH: Parathyroid hormone
MS: Mean square
PCA: Principal component analysis
